# Supplementary figures and images for: Salivary immune responses after COVID-19 vaccination
Source: PLoS One. 2024 Sep 3;19(9):e0307936. doi: 10.1371/journal.pone.0307936 (PMC11371244; doi:10.1371/journal.pone.0307936)

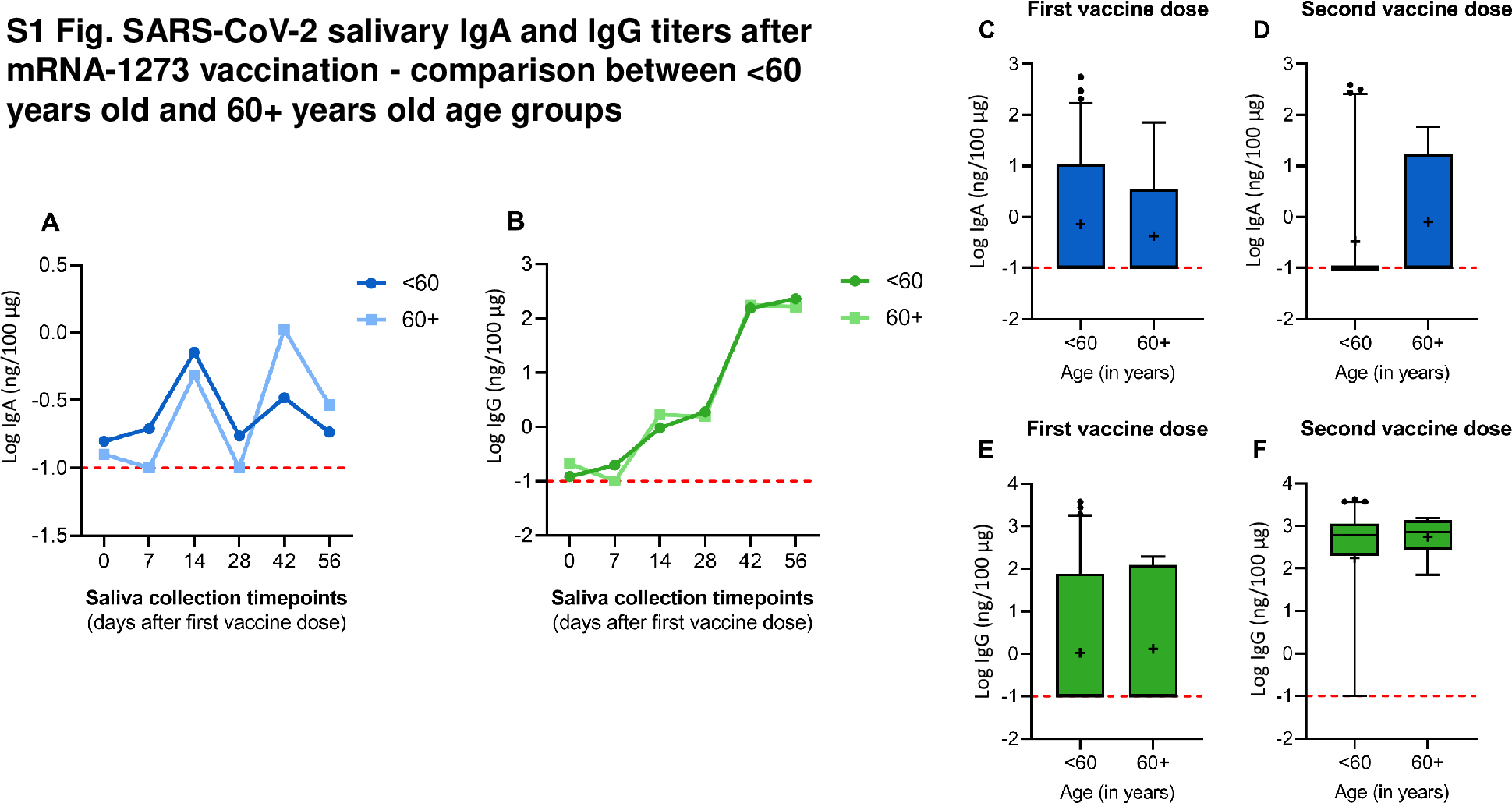

Supplement: S1 Fig — (TIF) [file pone.0307936.s002.tif]

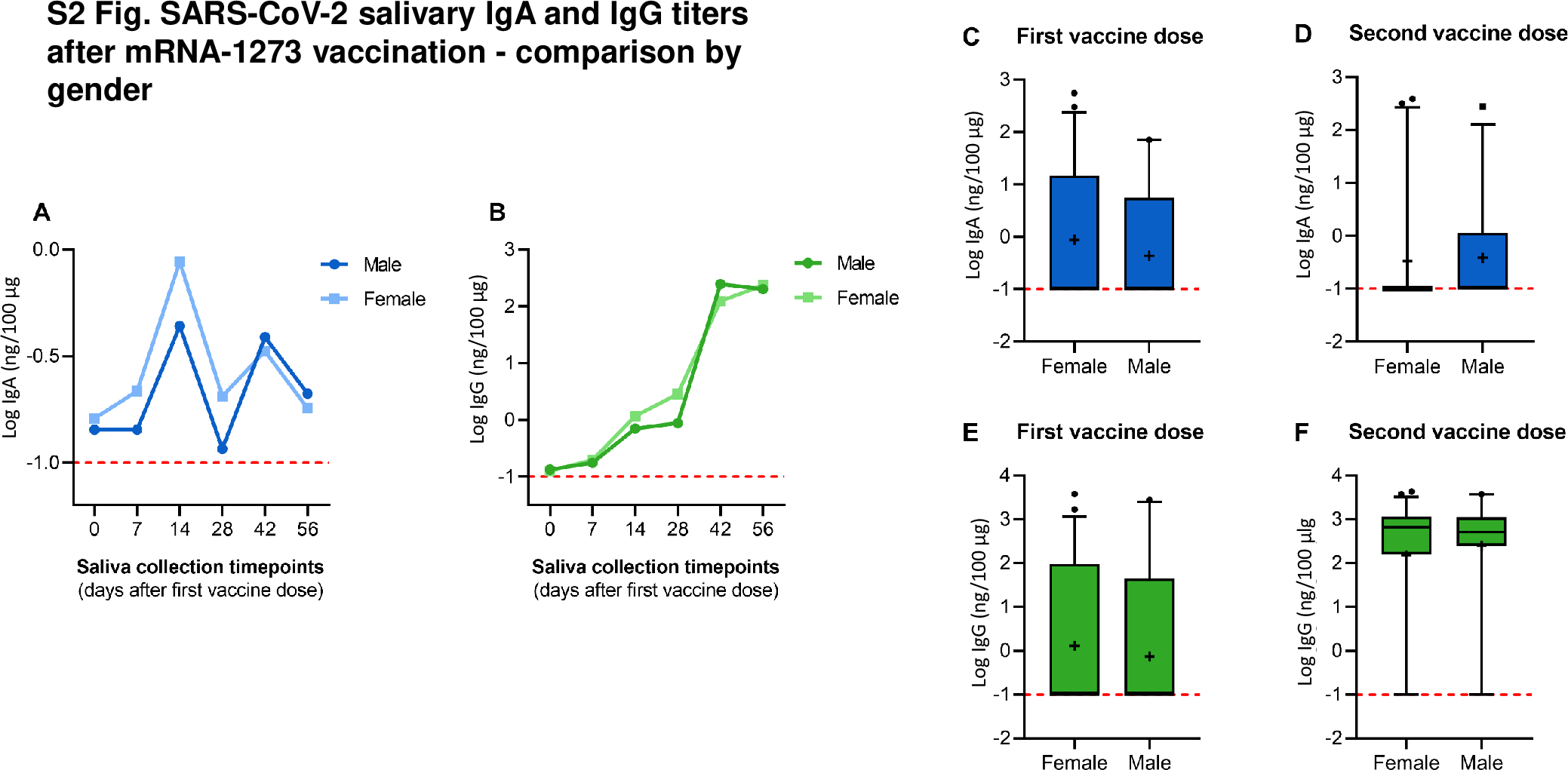

Supplement: S2 Fig — (TIF) [file pone.0307936.s003.tif]

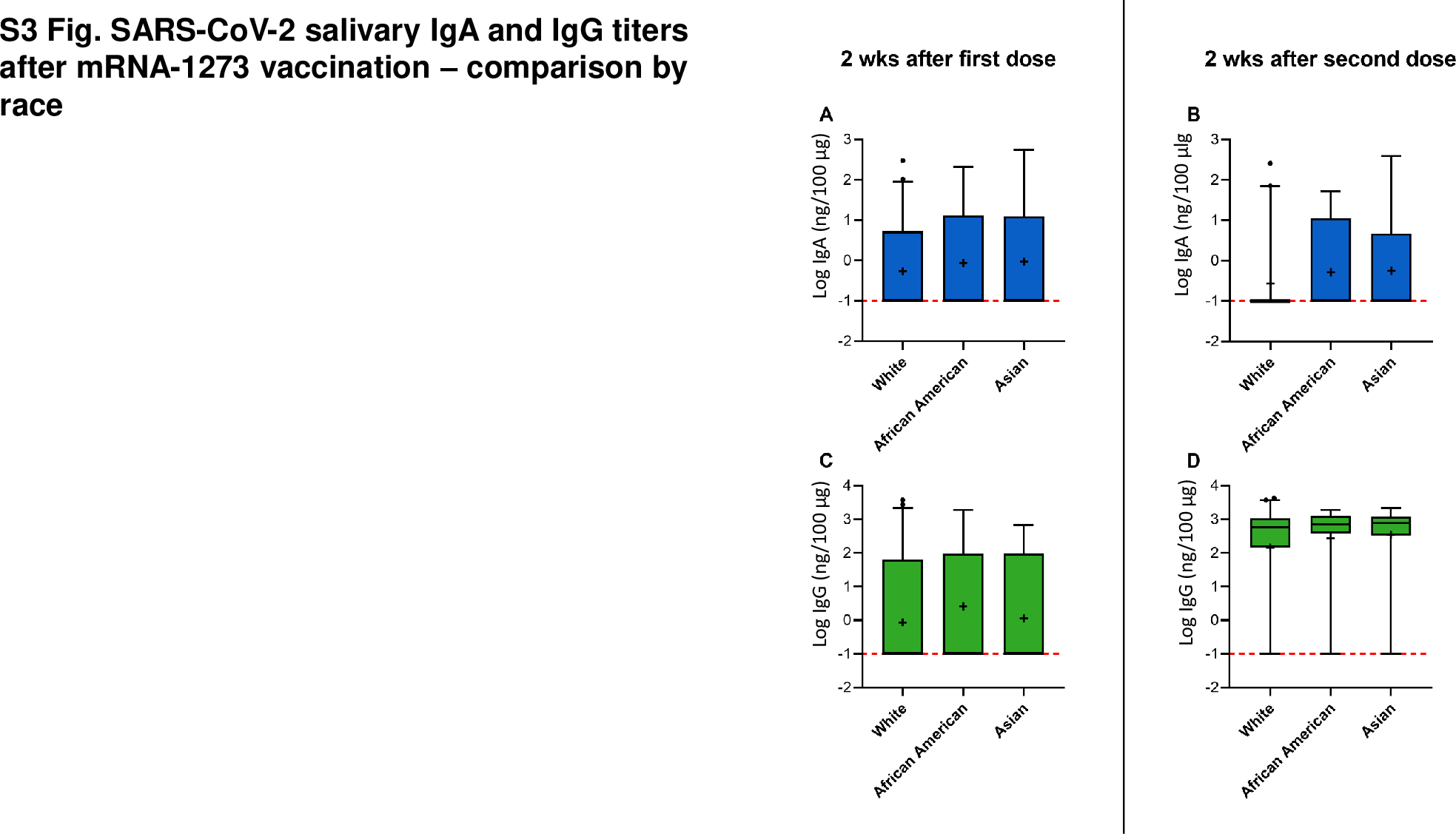

Supplement: S3 Fig — (TIF) [file pone.0307936.s004.tif]
