# Supplementary material for: Salivary immune responses after COVID-19 vaccination
Source: PLoS One. 2024 Sep 3;19(9):e0307936. doi: 10.1371/journal.pone.0307936 (PMC11371244; doi:10.1371/journal.pone.0307936)
Supplement: Attachment — Submitted filename: REBUTTAL manuscript PONE-D-23-39898.docx [file pone.0307936.s005.docx]

**RESPONSE TO EDITOR/REVIEWER COMMENTS**

We would like to thank the reviewers for their critical review of our manuscript and their helpful comments to more clearly describe the data leading to a significantly improved revised manuscript. Many thanks for your time!

**Reviewer #1**: This is an interesting study whose objective was to evaluate the salivary IgA and IgG immune response after vaccination with the COVID-19 Moderna mRNA-1273 vaccine. Their results confirm the potential utility of saliva in assessing immune responses after immunization and highlights that testing could lead to novel insight on mucosal immune response against SARS-CoV-2.

MINOR REVISION:

The infection status of the individuals before or after vaccination is not mentioned along the writing. Figure 4 is showing reactivity to nucleocapsid for some individuals and in Figure 3, saliva samples showed positivity at day 0 and 7, indicating that some patients had viral exposure within or before the study period. Because SARS-CoV-2 infections occurring before, between or after doses could influence the level and persistence of anti-SARS-CoV-2 IgA or IgG antibodies in saliva and blood specimens, it would be advisable to incorporate this information into the study. Additionally, it would be important to conduct analyses comparing individuals who experienced infections with those who did not, to recognize if there are any significant differences. Or at least justify why it is not shown.

**RESPONSE:** Healthy participants without respiratory symptoms were enrolled immediately after they received their first mRNA vaccine dose and although we asked if they had experienced symptoms that could be associated with COVID-19, most of them declined to provide that info and no nurses were available to collected a day 0 blood sample and hence the only lab data we have on their infection status is a saliva sample at day 0.

**Reviewer #2:** Dear authors, please address the following points:

**Comment** #1: - line 111 authors state they used pre-pandemic saliva samples as negative control, please clarify the source of these sample, where they came from a bank of saliva sample collected from generic patients before pandemic? How were they stored?

**RESPONSE:** Negative saliva samples were collected in 2009-2010 from healthy adults and stored at -80°C. We have modified to include this information and a reference to the original study (line 114 in the revised manuscript)

**Comment** #2-line 125 authors wrote: "participants self-collected whole blood", in my opinion it is worthy specify that the collected sample are Capillary blood sample;

**RESPONSE:** Thanks. We updated that capillary blood samples were collected.

**Comment** #3- line 132 remove one square bracket;

**RESPONSE:** bracket removed

**Comment** #4- line 174 the total number of 1023 seems wrong. (200-42)*6=948. If you excluded 42 patients from the original 200, it means you analyzed 158 patients. So the total number of collected samples should be 948.

**RESPONSE:** We have reviewed the data and realized that the statement in line 174 is incorrect. A total of 203 participants were enrolled, and 3 dropped out the study. Not all participants provided 6 samples (day 0-56). Overall, 1023 saliva samples were collected. Six months after the first dose, 23 participants provided an additional saliva sample. Hence, a total of 1046 saliva samples were collected, tested and analyzed. We have corrected the manuscript as follows:

Line 31: Two hundred was replaced as “…Two hundred three…”

Line 174 (line 118 in revised version): We replaced “200” by “203”

Line 175 (line 182 in revised version): We replaced “42” by ”3” and deleted the statement “or inability to complete the entire sample collection series through day 56.”

Line 176 (line 183-184): We have updated the sentence to reflect the number of saliva samples collected per day and the total number of saliva samples. “Overall, 1023 saliva samples were collected from 200 participants between day 0 and day 56. The median number of samples collected per day was 161.5 (range 155-200).”

Line 179 (185-186): rephrased to ”Additionally, 23 participants provided an extra sample 6 months after the first dose for SARS-CoV-2 specific G antibodies detection”

Figure 1: The 6 months’ time-point was included as well as the number of saliva samples collected for each time point. The legend was also updated.

**Comment** #5- line 178. In table 1 you should report information on the 158 patients really enrolled in the study excluding information of patients excluded.

**RESPONSE:** We are sorry for the misunderstanding. As explained for comment #4 above, data from 200 participants were analyzed.

**Comment** #6- line 186 The dot at the end of the line is a typo. A dot instead should be inserted at the end of line 187.

**RESPONSE:** Removed and added a dot were indicated.

**Comment** #7- line 191 Authors stated they collected blood samples from 127 participants, was this blood collected from a venipuncture procedure? Please clarify.

**RESPONSE:** “Blood samples was replaced by **“**Capillary blood sample”

**Comment** #8- line 194 and line 254. Authors analyzed data reporting median and percentile, meaning data are not distributed normally. But they performed ANOVA test, that is usually used for data distributed normally. Can authors please clarify and justify the statistical test performed?

**RESPONSE:** Upon reviewing your concern about the use of ANOVA for data reported as median and percentiles, we realized that the final analysis was conducted using ***unpaired t-tests***. The error in the manuscript was due to the fact that we initially attempted ANOVA analyses but later switched to non-parametric tests due to our data characteristics, sample size/distribution, and comparative analyses of the different sub-groups. Relevant sections have been corrected in the revised manuscript.

**Reviewer #3**: The manuscript reports induction of salivary antibody titers upon vaccination against SARS-CoV-2. Authors report significant increase in anti-Spike IgA and IgG two weeks after vaccination. It is a bit surprising, given that the development of antibody response takes around 2-3 weeks. This suggests that antibody response detected in these patients is driven by memory B cells, but not by naive B cells.

The data on the status of participants are required: were these donors previously infected? Figure 4 reports some of the data that can be used to determine the status, but these data are measured at day 42 after vaccination. It is of particular importance for the data interpretation, since we have recently shown that in non-exposed participants salivary immune responses develop much later (Bondareva et al., Cell Host and Microbe, 2023).

**RESPONSE:** see response to reviewer #1 above. Healthy participants without respiratory symptoms were enrolled immediately after they received their first mRNA vaccine dose and although we asked if they had experienced symptoms that could be associated with COVID-19, most of them declined to provide that info and no nurses were available to collected a day 0 blood sample and hence the only lab data we have on their infection status is a saliva sample from each participant collected at day 0.

Also, authors do not provide the levels of total IgA/IgG in saliva. These parameters may vary quite extensively in saliva during collection and the normalization to total IgA/IgG levels may further show whether this effect due to increase of specific antibodies or total antibody levels (Bondareva et al., J of autoimmunty, 2022).

**RESPONSE:** Thank you for pointing that out as we did normalize SARS-CoV-2 titers to total IgA and IgG and have now included additional language to the manuscript. Lines 157-160 in the revised manuscript. To account for participant differences (e.g., severity of illness, immunocompetency, collection time, antibody secretion levels), SARS-CoV-2 specific IgA or IgG was normalized to 100 µg of total IgA or IgG, respectively. When virus-specific antibodies could not be detected, but total IgA or IgG antibodies were detected, SARS-CoV-2-specific IgA (or IgG) were arbitrarily assigned as half the lower limit of detection, based on the standard curve of purified human IgA (IgG) (Costantini et al., 2022).

Minor comments:

Authors describe in the introduction the salivary Ab measurement as easier way to estimate blood Ig levels, but do not really mention that Ab in the oral cavity are of physiological significance, since they do actually protect against the initial virus infection. I would add this in introduction with the relevant quotes.

**RESPONSE:**  Thank you. Added to the introduction.

Authors do not cite a lot of relevant literature that was focused on salivary Ab responses, including series of works from J. Gommermann, and others. Thus, discussion should be extended and should include all the relevant papers published on this topic. So far it is quite poor and ignores many seminal works addressing the kinetic and importance of these responses.

**RESPONSE**: Thank you very much. We have added additional relevant papers on salivary antibody responses in the Discussion.
