# Supplementary material for: Salivary immune responses after COVID-19 vaccination
Source: PLoS One. 2024 Sep 3;19(9):e0307936. doi: 10.1371/journal.pone.0307936 (PMC11371244; doi:10.1371/journal.pone.0307936)
Supplement: Attachment — Submitted filename: Response to Reviewers PONE-D-23-39898R2.docx [file pone.0307936.s006.docx]

**RESPONSE TO EDITOR/REVIEWER COMMENTS – PONE-D-23-39898R2**

We would like to thank the reviewers for their critical review of our manuscript and their helpful comments to more clearly describe the data leading to a significantly improved revised manuscript. Many thanks for your time!

**Reviewer #2:**

Dear Editor, in my opinion the authors have addressed all the questions raised and now the paper is suitable for publication.

**Answer:** Thank you

**Reviewer #3**:

As it became evident during revision, the previous exposure to SARS-CoV-2 by participants cannot be ruled out. Thus, the conclusions about the kinetics of immune response upon vaccination cannot be made. In my opinion, this is a crucial point of the paper. Authors could have critically evaluated their data based on the already published reports, where clinical status was well documented. However, they also did not provide proper discussion in the revised manuscript and do not cite relevant literature.

**Answer:** We expanded on the limitation paragraph of our manuscript as we cannot rule out previous exposure to SARS-CoV-2 infection. We expanded on the data of the 9.4% of participants who tested positive for nucleocapsid antibodies 42 days after vaccination and thus the 90.6% who didn’t have serum antibodies, and emphasized that our conclusion on the kinetics of the immune responses after vaccination should be taken with caution and rephrased the paragraph as follows:

Our study has several limitations. First, since we only tested serum samples from a subset of 127 participants for nucleocapsid antibodies, previous exposure to infection with SARS-CoV-2 of the remaining 73 participants during the time of the study cannot be ruled out. We detected nucleocapsid antibodies in 9.4% at day 42 post vaccination, an indication of an infection either prior to the enrollment of the participant or during the study. Most published studies have been shown that nucleocapsid antibodies can be detected at least for 11 months after SARS-CoV-2 infection [33, 34]. However, recent data have shown that that in non-exposed participants salivary immune responses after SARS-CoV-2 infection may develop much later [35], which could have been addressed by testing blood samples beyond day 42 after vaccination. Therefore, our overall conclusions on the kinetics of the immune responses after vaccination should be taken with caution. Second, our study population was relatively small reducing the potential generalizability of the findings and limit their statistical power. In addition, our results do not represent the race or ethnicity distribution in the USA. We also did not consider other factors that may impact the salivary immune response to COVID-19 vaccination, such as the presence of comorbidities or use of certain medications that may affect immune functions or previous infection history [36]. Finally, we did not evaluate whether salivary antibodies did neutralize SARS-CoV-2, which could provide more conclusive information on immune protection against the virus [37, 38].
